# Mutational landscape of normal breast tissues adjacent to invasive breast cancer

**DOI:** 10.1016/j.xcrm.2025.102543

**Published:** 2026-01-08

**Authors:** Aleksandra Suwalska, Mariya Rozenblit, Malini Harigopal, Jiawei Dai, Meng Liu, Jeffrey P. Townsend, Michal Marczyk, Lajos Pusztai

**Affiliations:** 1 Department of Data Science and Engineering, Silesian University of Technology, 44-100 Gliwice, Poland; 2 Yale Cancer Center, Yale University, New Haven, CT 06510, USA; 3 Department of Pathology, Mount Sinai Hospital, New York City, NY 10029, USA; 4 Department of Biostatistics, Yale School of Public Health, New Haven, CT 06510, USA

**Keywords:** early-onset breast cancer, germline mutations, somatic mutations, mutational landscape, pathway enrichment analysis

## Abstract

Individuals with a history of breast cancer are at increased risk of developing a new breast cancer during their lifetime. Rare but high-impact somatic mutations in normal breast tissues may contribute to malignant transformation. We analyze mutations in cancer-relevant pathways across matched samples of peripheral blood, cancer-adjacent normal breast, and breast cancer from patients diagnosed before 50 years of age who carry no germline mutations in cancer-predisposing genes. Gene- and pathway-level mutation profiles and single-base substitution (SBS) signatures are compared between tissue types in two independent cohorts (Yale, *n* = 24; TCGA, *n* = 17). Cancer-adjacent normal breast tissue contains multiple acquired somatic mutations that persist in tumors. Most variants are shared across tissue types from the same individual, indicating strong germline influence. The substantial germline contribution to alterations through common and rare polymorphisms in cancer hallmark pathways supports a model of cancer risk based on the collective impact of variants in cancer-related genes.

## Highlights

• BC patients harbor many protein-altering germline variants in cancer-relevant genes
• Cancer-adjacent normal breast tissues carry many of the same mutations found in cancer
• A patient-specific pattern of pathway-level mutations indicates phenotypic convergence
• Vast gHFIs not linked to cancer risk suggest context-dependent biological effect

## Graphical abstract

**Figure:**
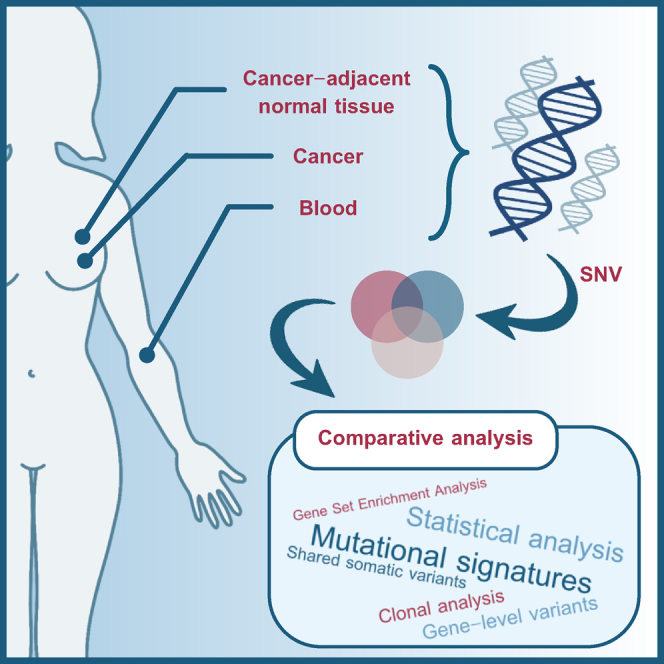

## Teaser

Suwalska et al. show that cancer-adjacent normal breast tissues share genomic alterations with cancer, and many changes in cancer-relevant genes arise from specific combinations of germline polymorphisms. This supports gradual mutation-driven malignant transformation of normal cells and suggests context-dependent co-oncogenic function for common and rare germline polymorphisms.

## Introduction

Breast cancer has emerged as the predominant cancer diagnosis and primary cause of cancer-related mortality in women in 107 out of 184 countries based on recent World Health Organization estimates.^1^ It is a complex and multifactorial disease with a pathophysiology that remains incompletely understood. The development of breast cancer is believed to involve a combination of environmental, lifestyle, hormonal, and genetic factors. Advancing age and being female are the most common risk factors associated with the disease^2^; however, the incidence of early-onset breast cancer, diagnosed in young women before the age of 50 years, is increasing.^3^ Early-onset breast cancer is characterized by larger tumor size, more frequent lymph node involvement, less differentiation, and more frequent lack of estrogen receptor expression compared to breast cancers in older women.^4^^,^^5^ Overall, less than 10% of early-onset breast cancers are attributable to germline BRCA1 or BRCA2 mutations,^3^ and more than 80% of patients with early-onset breast cancer do not have any known cancer-predisposing germline mutations.^4^ Nevertheless, personal history and family history of breast cancer are risk factors for developing breast cancer even in the absence of germline mutations in high-penetrance cancer genes.

Mutations in genes with no significant association with cancer risk can still have a role in cancer pathogenesis due to genomic context-dependent biological effects. In all cancers, the biological changes necessary for malignant transformation result from a complex interplay of accumulating acquired somatic mutations and common and rare high functional-impact germline polymorphisms (gHFI) in cancer-relevant genes.^5^ Even classical oncogenes cannot on their own transform a normal cell into a malignant cell, indicating that malignant transformation requires alterations in many cellular processes referred to as hallmark pathways of cancer.^6^ The spectrum of cancer-relevant genes is broader than previously thought,^5^^,^^7^^,^^8^^,^^9^ and many rare somatic mutations can have functional importance in cancer biology.

We have previously shown that cancer-adjacent normal breast tissues, while histologically appearing normal, show evidence of accelerated epigenetic aging.^10^^,^^11^ There is growing evidence that both genetic and epigenetic field defects in normal or peritumoral breast tissue exist and forecast future breast cancer risk. Widespread cancer-associated DNA methylation patterns in histologically normal breast tissue have been observed, suggesting epigenetic field effects that predate cancer formation.^12^ Analysis of TCGA data showed that 40% of cancer-adjacent, benign-appearing tissues harbored genomic defects in DNA copy number, sequence, methylation, or RNA sequence.^13^ The concept that tumor-adjacent normal breast tissue may harbor cancer-predisposing molecular alterations is also supported by clinical observations. Radiation therapy of the breast after lumpectomy reduces the risk of developing a new primary tumor in the same breast,^14^ suggesting that breast epithelial cells with normal morphology can carry alterations that increase their chance for future malignant transformation. Therefore, we hypothesized that in women with a high-risk family history, normal breast tissue adjacent to breast tumor already harbors germline and somatic mutations that may have contributed to the development of breast cancer.

In this study, we analyzed whole-exome sequencing (WES) data from two independent cohorts of women diagnosed with breast cancer at ages younger than 50 years and assessed germline and somatic mutation patterns in paired triplet tissue samples of blood, cancer-adjacent normal breast, and cancer.

## Results

### Somatic and high-functional impact variant burden across tissue types

The average numbers of exonic, non-silent variants in the Yale cohort were 10,885 per sample in the blood (range: 10,341–12,582), 10,836 in cancer-adjacent normal (range: 8,385–12,774), and 11,159 in cancer tissue (range:10,532–12,779). In the TCGA cohort, the corresponding numbers were 9,744 (range: 8,033–12,703), 9,862 (range: 8,505–12,700), and 9,718 (range: 8,220–12,939) for blood, normal breast, and cancer tissues, respectively. After filtration for high-functional impact variants, the average number of variants in the Yale cohort decreased to 171 (range: 140–212) for blood samples, 174 (range: 138–213) in cancer-adjacent normal, and 191 (range: 154–270) in cancer samples. The average numbers of high-functional impact variants in TCGA samples were 177 (range: 141–239), 184 (range: 149–277), and 199 (range: 141–350) in blood, normal breast, and cancer tissues, respectively. Venn diagrams showing the overlap of affected genes (Tables S2 and S3) in paired samples of the three tissue types for each patient are shown in Figure S2 for the Yale and TCGA cohorts, respectively. Most of the mutated genes are shared between all three sample types, indicating a large germline contribution (Table S4).

### Clonal architecture and phylogenetic relationships between tissues

To assess the clonal representation of somatic mutations within cancer-adjacent normal tissues, we analyzed the variant allele frequencies (VAFs) of all somatic mutations identified in this tissue type. VAFs ranged from 0.067 to 0.637, with a median of 0.216 and an interquartile range (IQR) of 0.117, indicating substantial heterogeneity in clonal abundance across variants. These values suggest that while some mutations are likely confined to small subpopulations of cells, others may be present in a substantial fraction of epithelial cells in the sampled regions (Figure S4B).

**Figure 1:**
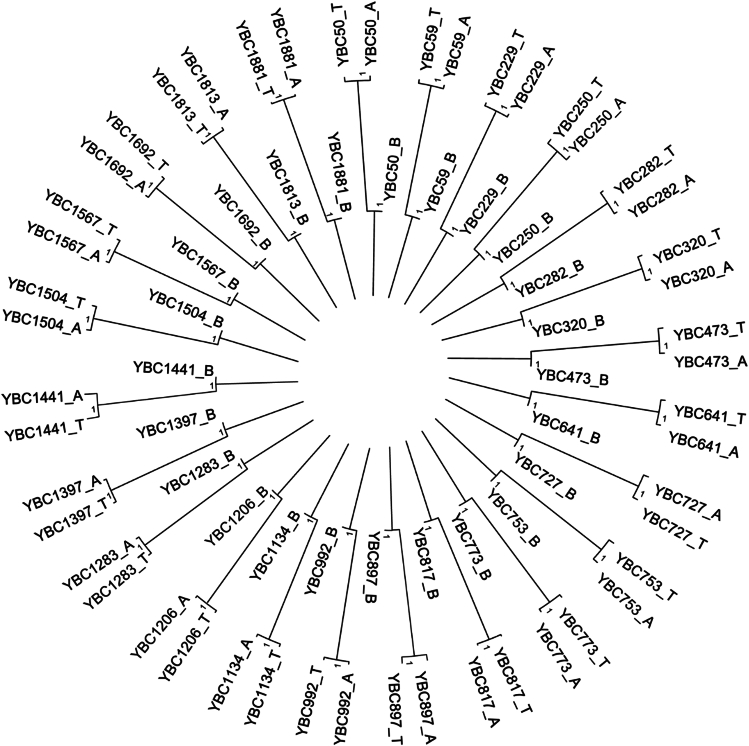
Phylogenetic relationships among 24 lung cancer patients inferred from BEAST2 maximum clade credibility (MCC) trees Each uncalibrated chronogram represents the evolutionary relationship among tumor (T), adjacent normal (A), and blood (B) samples from an individual patient. Phylogenies were inferred using BEAST2 and are displayed radially around a circular layout for visualization. The blood (B) sample represents the germline sequence and is positioned toward the inner circle, while tumor (T) and adjacent normal (A) samples are aligned along the outer circle, reflecting their relative evolutionary divergence from the germline. Posterior probabilities are shown on internal branches. See also Figure S3.

To evaluate clonal relationships between cancer-adjacent normal tissue and tumor, we reconstructed per-patient phylogenies based on validated somatic variants jointly detected across blood (B), adjacent normal (A), and tumor (T) samples (Figures 1 and S3). Across all 24 patients, the blood sample (constituting the germline sequence) was consistently placed at the root. Cancer-adjacent normal tissue and tumor samples were closely related with very limited divergence, indicating extensive shared somatic ancestry.

### Distribution and sharing of somatic mutations in normal and tumor tissues

The distribution of somatic mutations across tissue types for each patient was quantified, classifying them as private to the tumor, private to normal, or shared (Figure S4A). Shared somatic variants were further analyzed for their VAFs in both tissues (Figure S4C). The proportion of shared mutations varied across patients, and VAF comparisons indicated that some alterations may arise early and expand clonally in both tissues. For shared somatic variants, the mean tumor-to-normal VAF ratio was 1.05 (range = 0.59–2.0), indicating that shared mutations exhibited comparable allelic representation across tissues, consistent with early clonal seeding rather than later contamination. The list of shared somatic mutations for the Yale cohort with details, VAFs, and damaging predictors (CADD, MetaSVM, ClinPred) is included in Table S5.

Several recurrently altered genes, including SUSD2, PABPC3, PRSS1, ZNF717, and SVIL, were observed in both cancer-adjacent normal and tumor tissues. These variants carried high CADD scores (≥20) and were consistently classified as deleterious by MetaSVM and ClinPred. Variant allele frequencies further indicate substantial clonal representation in both tissue types, for example, SUSD2 (VAF 0.69 in normal vs. 0.83 in tumor), SVIL (0.47 vs. 0.72), and TAS2R30 (0.33 vs. 0.44). Importantly, TCGA RNA sequencing (RNA-seq) data revealed that several of these genes, like SVIL (log2FC −0.66, FDR 0.003) and TAS2R30 (log2FC −0.10, FDR <0.001) exhibit significant differential expression between tumor and adjacent normal tissues. Together, these examples demonstrate that shared high-impact variants are not only predicted to impair protein function but also coincide with measurable transcriptional changes, underscoring their possible contribution to malignant transformation.

### Frequently mutated genes in Yale and TCGA cohorts

**Figure 2:**
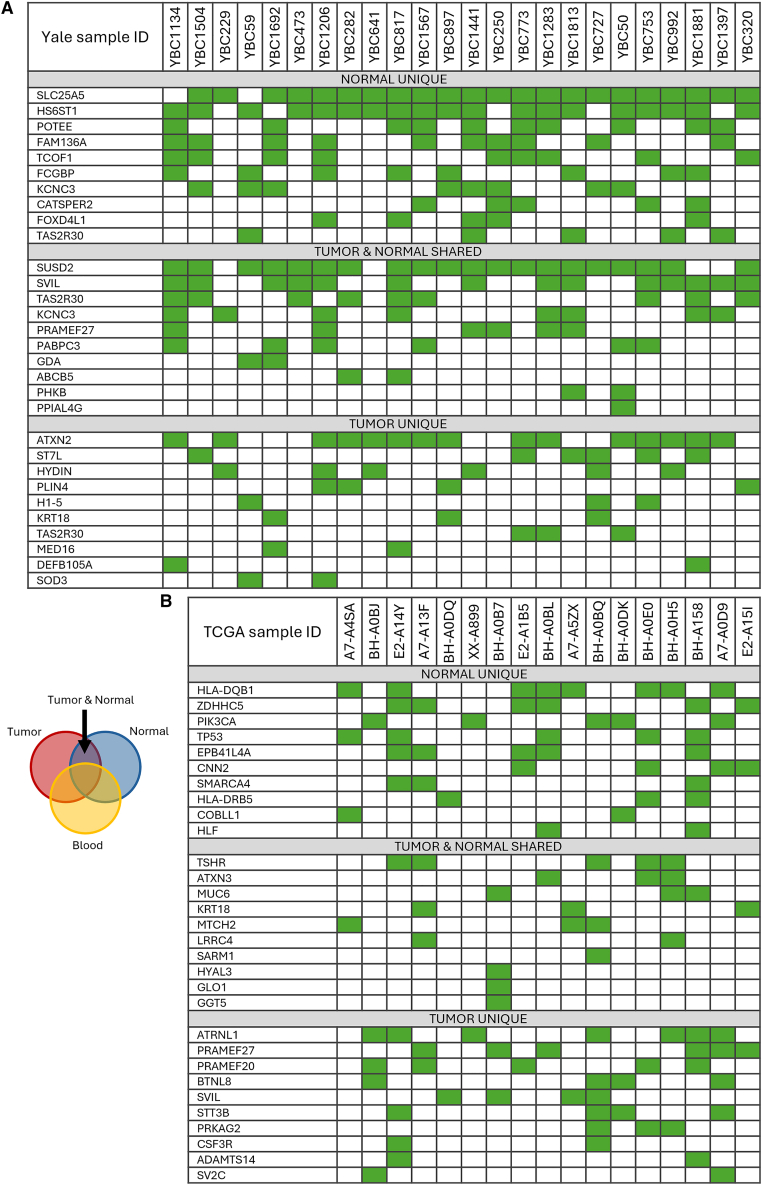
Somatically mutated genes that are shared across or specific to breast cancer and tumor-adjacent normal breast tissues Each column represents a patient, and each row represents a gene. The green squares indicate mutations, and the white squares correspond to wild-type genes. (A) Yale cohort. (B) TCGA cohort. See also Tables S2, S3, and S4 and Figure S3.

The top 10 most frequently somatically mutated genes in the cancer-adjacent normal and cancer tissues contain some that are shared by Yale and TCGA datasets (Figure 2). The most frequently somatically mutated genes in cancer in the Yale cohort were SLC25A5 (91.67% of patients) and HS6ST1 (79%), in cancer-adjacent normal tissue it was ATXN2 (62%), and the most frequently mutated genes shared between paired cancer and cancer-adjacent normal were SUSD2 (83%) and SVIL (58%). In the TCGA, the most frequently somatically mutated gene in breast cancer tissue was HLA-DQB1 (47% of patients), in cancer-adjacent normal tissue ATRNL1 (41%), and in both TSHR (26%). These differences are likely due to differences in statistical power to identify differentially mutated genes in the two small cohorts and differences in sequencing coverage (in TCGA ∼100× for tumor, normal breast, and blood; in the Yale cohort ∼100× for blood and normal; and ∼200× for cancer; Figure S1 A).

### Mutational signatures across tissues and cohorts

The mutational signature analysis identified three COSMIC signatures, including SBS1, caused by spontaneous deamination of 5-methylcytosine that correlates with aging, and SBS5, an age-associated signature of unknown etiology. These observations are consistent with prior reports that found epigenetic signals of accelerated aging in normal breast tissues of women with breast cancer.^10^^,^^11^ The third signature, SBS54, has an unknown etiology and could be caused by sequencing artifacts. The percent contributions of these signatures were similar across the sample types and datasets, without any statistically significant difference (Table 1; Figure S5).

**Table 1:** The number and percent of variants in each tissue type and dataset affected by a given mutational signature

	Blood		Normal		Tumor	
Signature	Yale	TCGA	Yale	TCGA	Yale	TCGA
SBS1	103,736 (19.1%)	63,221 (18.5%)	102,048 (19.0%)	62,828 (18.5%)	104,751 (18.8%)	62,113 (18.4%)
SBS5	317,118 (58.4%)	205,418 (60.2%)	316,784 (58.9%)	205,486 (60.4%)	328,642 (59.1%)	203,116 (60.3%)
SBS54	122,070 (22.5%)	72,695 (21.3%)	119,358 (22.2%)	71,982 (21.2%)	123,153 (22.1%)	71,431 (21.2%)

See also Table S1; Figures S1 and S5.

### Differentially mutated genes between tissues

**Figure 3:**
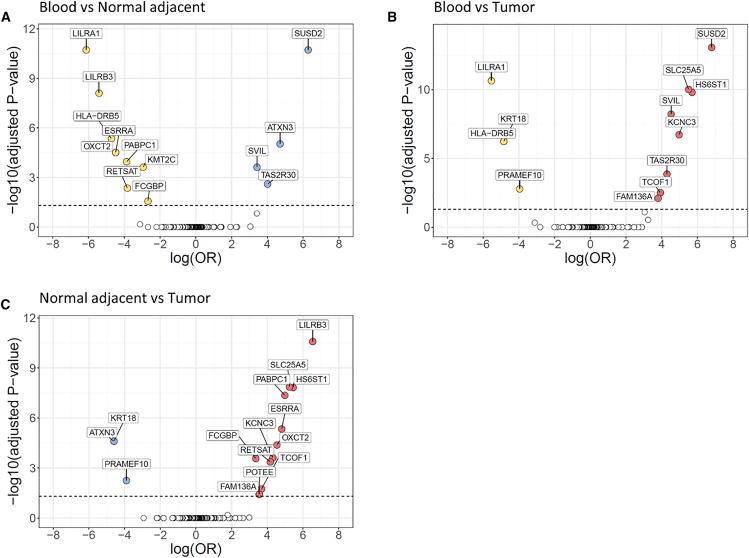
Differentially mutated genes between sample types in the Yale cohort The horizontal line indicates the *p* = 0.05 significance threshold (Fisher’s exact test). Effect size is determined as the logarithm of the odds ratio (*x* axis). The color indicates in which tissue the gene is mutated more frequently. (A) Blood (yellow) vs. normal adjacent (blue). (B) Blood (yellow) vs. tumor tissue (red). (C) Normal adjacent (blue) vs. tumor tissue (red). See also Tables S6 and S7.

In the Yale cohort, comparing mutation frequencies between blood versus tumor-adjacent normal breast tissue resulted in 13 significantly differentially mutated genes. The comparison of blood versus breast cancer resulted in 12 genes, and a comparison of tumor-adjacent normal versus breast cancer identified 15 differentially mutated genes after the correction for multiple testing (Figure 3; Table S6). In TCGA, we found no differentially mutated genes between the three types of samples after adjusting for multiple comparisons, probably due to limited power in each cohort and coverage differences between the cohorts (Table S7).

### Pathway-level alterations driven by somatic and germline variants

**Figure 4:**
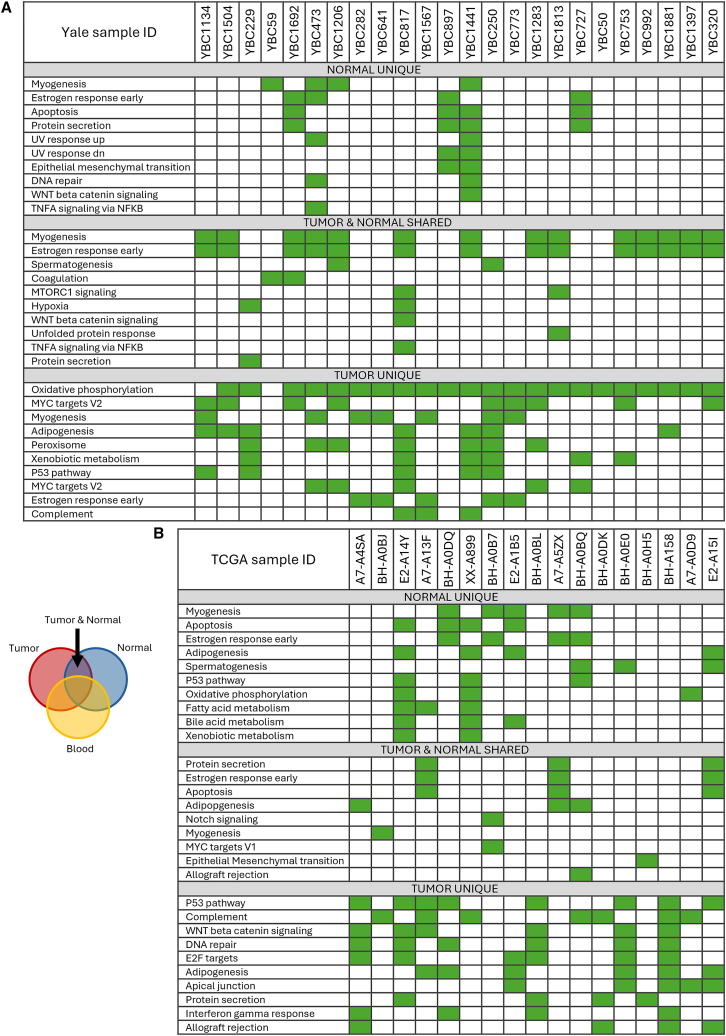
Patient-level cancer hallmark pathway alterations in the paired cancer-adjacent normal breast and breast cancer Each column represents a patient, and each row represents a pathway affected by at least one somatic mutation unique to the normal, or tumor tissues, or shared between them. The green color indicates the affected hallmark pathway. (A) Yale cohort. (B) TCGA cohort. See also Figures S6 and S7.

**Figure 5:**
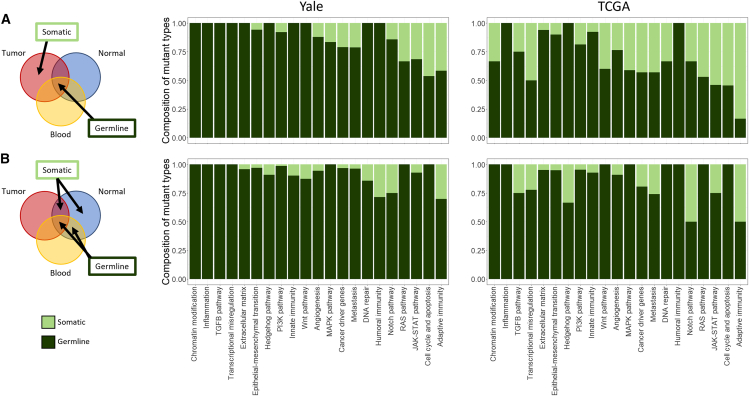
Contribution of germline variants (dark green) and somatic mutations (light green) to pathway alterations in cancer-adjacent normal breast tissues and breast cancers The bars indicate the percent of affected genes in a given pathway with germline and somatic origins. (A) Somatic mutations are defined as variants found in the tumor only and germline variants are defined as shared variants by all tissue types from a given patient in the Yale and TCGA cohorts. (B) Somatic mutations are defined as variants found in normal tissue only and shared between normal and tumor, while germline variants are defined as shared by all tissue types and shared between blood and normal. See also Table S5 and Figure S4.

When gene-level alterations were mapped into Cancer Hallmark pathways, most pathway alterations were due to cancer-specific somatic mutations; however, many of the same pathways were also affected by somatic mutations in cancer-adjacent normal tissues (e.g., myogenesis, estrogen response, >50% of patients) (Figure 4). Tumor tissues showed the highest frequency of pathway mutations in both Hallmark and KEGG collections. In the Yale cohort, the most frequently affected KEGG pathway (>50% of patients) was the calcium signaling pathway, while in the TCGA cohort it was the pathways in cancer. Cancer-relevant pathways were therefore affected by somatic mutations in cancer-adjacent normal tissues in almost all patients. Figure S6 shows the results of pathway mutation enrichment analysis for the KEGG cancer pathways. The GSEA results for KEGG pathways are presented in Figure S7. In the Yale dataset, only the peroxisome pathway was significantly enriched (FDR-adjusted) in blood and tumor samples relative to cancer-adjacent normal breast tissues. In the TCGA, the E2F pathway was enriched in mutations in tumors compared to normal samples. Overall, germline variants were the main source of pathway alterations in almost all pathways; they were particularly dominant in chromatin modulation, inflammation, TGF-beta signaling, DNA repair, and humoral immunity pathways (Figure 5). Somatic mutations contributed most frequently to alterations in the cell-cycle, JAK-STAT, and adaptive immunity pathways. There were substantial variations across the datasets in the percentage of genes affected by germline variants versus somatic mutations in any given pathway. This is likely due to the small sample sizes of both cohorts, which limit the precision of the estimates.

**Figure 6:**
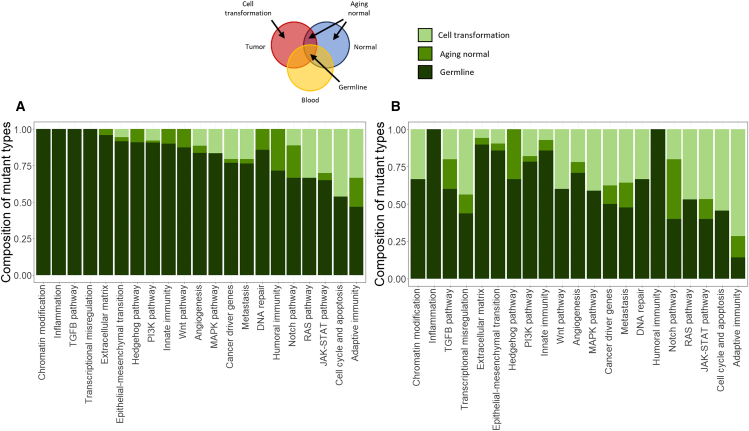
The average percent contribution of germline polymorphisms versus somatic mutations to alterations in Cancer Hallmark pathways The bars indicate the percent of affected genes in a given pathway with germline and somatic origins, respectively. (A) Yale cohort. (B) TCGA cohort.

We also assessed the joint proportion of mutations in cancer hallmark pathways detected in blood, normal breast tissue, and breast cancer (Figure 6). Germline variants were defined as those present in all paired tissues for a given patient, and somatic mutations included those present in either the cancer or the normal breast tissue but absent from the blood. Variants of germline origin contribute the most to gene-level alterations in all biological pathways. The somatic variants affected adaptive immunity, cell-cycle, apoptosis, and the JAK-STAT pathways most frequently.

## Discussion

We analyzed whole-exome sequencing data of paired cancer, cancer-adjacent normal breast tissue, and peripheral blood from women diagnosed with early-onset breast cancer. Our goal was to assess the extent and source of genomic alterations in cancer hallmark pathways in these tissue triplets. We found that each sample harbored from 138 to 350 predicted high-functional impact somatic and germline variants that could alter protein function in a large number of cancer-relevant genes. This study extends the results of earlier publications that have shown shared mutations in cancer and adjacent normal tissues^5^ to individuals with early-onset breast cancer and a strong family history. Our results draw attention not only to the shared somatic mutations between normal breast tissue and cancer but also to the very large number of germline variants that alter cancer-relevant pathways. This illustrates that cancer-relevant pathways are altered through unique combinations of genes in each person and challenge the dominant view that a few oncogenes drive malignant transformation.

Many of the somatic alterations were non-recurrent and unique to patients; the germline alterations represented common or rare polymorphisms. Non-recurrent somatic mutations are often relegated to passenger mutation status, and common germline polymorphisms are also usually not considered as biologically relevant when cancer genomic alterations are reported, and therefore, investigators often assume that only a few driver genes determine cancer biology.^15^^,^^16^ However, even classical oncogenes require additional mutations to transform cells, and their effects are cell-type and genomic-background dependent.^17^ Clinical observations also indicate that histologically very similar cancers have unique clinical courses with highly variable mutation patterns, treatment responses, and differences in time to progression and overall survival. We propose that the unique clinical behavior of each cancer may be determined by the combined effect of the large number of “passenger mutations” and germline alterations in cancer-relevant genes.^18^

The detection of shared somatic mutations between tumor and adjacent normal breast tissue across all patients suggests that clonal expansions may occur prior to histological transformation. A recent report showed that latent cancer cells carrying causal mutations can be stimulated to form tumors by exposure to tumor promoters or by chronic tissue damage in a mouse model.^19^ These results provide experimental support to our hypothesis that many cancer-adjacent morphologically normal-appearing cells are latent cancer cells that could transition to full malignant transformation with additional tissue insults. The observed variability in VAFs across tissue types and patients supports a model in which certain early alterations may reflect field effects or pre-malignant clonal evolution.

When we mapped gene-level alterations into biological pathways, many cancer-biology-relevant pathways were affected through unique combinations of somatic and germline events. Many of the pathways involving cell-cycle regulation, JAK-STAT signaling, and adaptive immunity were affected by cancer-specific somatic mutations, but often the same pathways were also affected by somatic mutations (in different genes) in cancer-adjacent normal tissues. When germline variants were also considered, due to the large number of protein-function-altering germline polymorphisms, the main source of pathway alterations was germline. The most frequently germline-altered pathways included chromatin modulation, inflammation, TGF-beta signaling, DNA repair, and humoral immunity pathways. These findings are consistent with several other reports that showed frequent somatic mutations in cancer-relevant genes in cancer-adjacent normal tissues, including mutations that are shared between normal and cancerous tissues.^13^^,^^20^^,^^21^ Collectively, these results indicate that histologically normal breast tissues already harbor some of the hallmark alterations of cancer. Biological “priming” of seemingly normal breast epithelial cells in patients with breast cancer is also supported by the clinical observation that individuals with a history of breast cancer have a high risk of developing a new breast cancer in the same or contralateral breast.^22^

The clinical relevance of the large number of germline variant-driven alterations in cancer hallmark pathways is unclear, but their functional importance in cancer risk is consistent with clinical observations. In an earlier study, we performed whole-exome sequencing to examine germline and somatic alterations in histologically normal breast tissues from women who subsequently developed breast cancer and compared these with results from breast tissues of women who did not at a median follow-up of over 6 years. The average number of somatic mutations was similar between the two cohorts, but patients who subsequently developed breast cancer had significantly more high-functional-impact germline variants than the healthy controls. Many of these were involved in regulating cell growth and genome instability. These results suggest that individuals who develop breast cancer have subtle deficiencies in cancer regulatory networks that, in the context of other germline and somatic mutations, could facilitate malignant transformation.^23^

Approximately one in three individuals will develop some form of invasive cancer during their lifetime in the United States, and most of these cases are not related to high-level carcinogen exposure genes.^24^^,^^25^ Family history of cancer remains an important and consistent risk factor for all cancer types, even though only a small minority of patients with a family history carry a known high-penetrance cancer predisposing germline variant.^26^ We previously showed that histologically normal breast tissues carry genomic changes related to cancer development years before a cancer diagnosis, and pre-diagnosis tissues had significantly more high-functional impact germline variants per sample than breast tissues from women who never developed breast cancer.^23^ We propose that the large number of germline variants with individually small latent effects may, in combination, create a genetic background that modulates the pace of malignant transformation. A latent effect refers to an alteration that does not result in immediate phenotypic change but renders a regulatory network more vulnerable to subsequent epigenetic or genetic hits. The contribution of this type of germline variants, which are not individually associated with cancer risk because they are context dependent, is missed by all polygenic GWAS risk models by design. The clinical implication of our results is that it lays the conceptual foundation of developing new cancer risk models that take into account all protein-altering alterations in cancer-relevant genes in germline and “normal” tissue biopsies.

Our study has some limitations. Our study population includes mostly young and Caucasian women; to what extent the results apply to older and more diverse populations still needs to be studied. The major limitation is that our sample sizes in both cohorts are small. Thus, it is not surprising that gene-level findings have limited overlap between the two datasets since the small sample sizes limit the precision of frequency estimates, and the analytical (fresh-frozen tissue in the TCGA, formalin-fixed paraffin-embedded tissues in the Yale cohort) and sequencing depth differences imply different sensitivities to detect alterations. Also, in the Yale cohort, the coverage was lower for normal tissues than for cancer, which could limit the detection of rare clonal variants in normal breast tissue. Given the requirement of at least 10 supporting reads per variant, the minimal detectable VAF was roughly estimated as 0.10 in normal and 0.05 in tumor datasets. Consequently, low-frequency variants (VAF <0.10) may be underdetected in normal breast tissues, leading to partial underrepresentation of rare clonal variants.

However, at a higher level, the results are consistent in showing frequent somatic mutations in cancer-adjacent normal tissues and large contributions to pathway alterations from germline source. Our results are descriptive, and the functional impact of variants and mutations is inferred from prediction algorithms and not validated in experimental systems, so some of our functional predictions may overestimate impact.^27^ It is practically impossible to experimentally validate the millions of predicted high-functional impact germline variants that the human genome has. On the other hand, we did not consider DNA structural variants and epigenetic changes that also regulate gene function, and therefore, the true extent of cancer hallmark pathway disturbances from combined somatic and germline origin is an underestimation in our study.

In summary, in triplets of peripheral blood, cancer-adjacent normal breast tissue, and breast cancer, whole-exome sequencing revealed extensive genomic alterations in cancer-adjacent normal tissues. Cancer hallmark pathways were affected by somatic mutations in cancer-adjacent normal tissues in almost all patients, and we also detected larger numbers of germline variants that could also affect these pathways. Shared somatic mutations in cancer and adjacent normal tissues were observed in all cancers. These germline variants and acquired somatic mutations are present in unique combinations in genes of cancer hallmark pathways in each patient. The large proportion of germline contribution to cancer hallmark pathway alterations through common and rare polymorphisms not linked to cancer risk suggests that a new model of cancer risk could be built by considering the totality of variants in cancer-related genes.

### Limitations of the study

Limitations of this study arise primarily from the relatively small sample size and limited demographic diversity, since participants were predominantly young Caucasian women, which restricts the generalizability of the findings to broader populations. Differences in sample processing and sequencing depth between the TCGA and Yale cohorts introduced variability in detection sensitivity and likely contributed to the underestimation of low-frequency clonal variants in normal tissues, particularly given the minimal detectable VAF of approximately 0.10 in normal samples. The analysis is descriptive and relies on computational predictions of functional impact without experimental validation, which may overestimate the biological relevance of some variants. Furthermore, structural variants and epigenetic alterations were not assessed, implying that the true extent of cancer hallmark pathway perturbations caused by combined somatic and germline changes is likely greater than reported here.

## Resource availability

### Lead contact

Requests for further information and resources should be directed to and will be fulfilled by the lead contact, Aleksandra Suwalska (aleksandra.suwalska@polsl.pl).

### Materials availability

This study did not generate new unique reagents or cell lines. All human tissue samples were obtained from the Yale Cancer Prevention Clinic (Yale University) and The Cancer Genome Atlas (TCGA) under appropriate institutional review board approvals and cannot be redistributed.

### Data and code availability

• Whole-exome sequencing data supporting the conclusions of this article is available in the Sequence Read Archive repository, BioProject number PRJNA1087680 (https://www.ncbi.nlm.nih.gov/sra/PRJNA1087680).
• Publicly available TCGA data used in this study can be accessed through the Genomic Data Commons (https://portal.gdc.cancer.gov/).
• Custom analysis scripts used for analysis are publicly available on Zenodo and can be accessed at DOI: https://doi.org/10.5281/zenodo.17675928.
• Any additional information required to reanalyze the data reported in this paper is available from the lead contact upon request.

## Acknowledgments

This research was partly supported by the 10.13039/100001006Breast Cancer Research Foundation investigator award (BCRF-22-133) and a Susan Komen Leadership grant (SAC220225) to L.P. and ASCO YIA 2020 awarded to M.R. A.S. was awarded with the SUT Excellence Initiative – Research University grant (32/014/SDU/10-27-05). This research was funded in part by the National Science Centre, Poland, grant no. 2023/50/E/NZ2/00583 (M.M.).

## Author contributions

Conceptualization, L.P. and M.R.; supervision, L.P., M.M., and J.P.T.; data acquisition and specimen preparation, M.R. and M.H.; data preprocessing and data analysis, A.S., J.D., and M.L.; data interpretation, A.S., M.M., L.P., M.R., and M.L.; writing—original draft, A.S. and M.R.; writing—review and editing, L.P., M.M., J.D., and A.S. All authors read and approved the final manuscript.

## Declaration of interests

L.P. has received consulting fees and honoraria for advisory board participation from Pfizer, Astra Zeneca, Merck, Novartis, Bristol-Myers Squibb, Stemline-Menarini, GlaxoSmithKline, Genentech/Roche, Personalis, Daiichi, Natera, and Exact Sciences and institutional research funding from 10.13039/100020124Seagen, 10.13039/100004330GlaxoSmithKline, 10.13039/100004325AstraZeneca, 10.13039/100004334Merck, 10.13039/100004319Pfizer, and 10.13039/100002491Bristol Myers Squibb.

## STAR★Methods

### Key resources table

**Table:** 

REAGENT or RESOURCE	SOURCE	IDENTIFIER
**Biological samples**		

Peripheral blood, breast tumor, and cancer-adjacent normal breast tissue samples	Yale Cancer Prevention Clinic (YCPC)	N/A
Human breast cancer cohort data	The Cancer Genome Atlas (TCGA)	N/A

**Chemicals, peptides, and recombinant proteins**		

Qiagen AllPrep Universal Kit	Qiagen	N/A
NEBNext FFPE DNA Repair Mix	New England Biolabs	Cat# M6630L
AMPure XP Magnetic Beads	Beckman Coulter	N/A
IDT xGen Exome Research Panel v1.0	Integrated DNA Technologies	N/A
Covaris E210 Sonicator	Covaris	N/A
Agilent 2100 Bioanalyzer	Agilent Technologies	RRID: SCR_018043
Illumina HiSeq 4000 System	Illumina	RRID: SCR_016386

**Deposited data**		

Whole-exome sequencing data (Yale cohort)	NCBI Sequence Read Archive (SRA)	BioProject: PRJNA1087680
TCGA breast cancer sequencing data	The Cancer Genome Atlas (TCGA)	dbGaP: phs000178.v11.p8

**Software and algorithms**		

Burrows–Wheeler Aligner (BWA)	Li et al.^28^	https://bio-bwa.sourceforge.net/
Picard Toolkit	Broad Institute	https://broadinstitute.github.io/picard/
Genome Analysis Toolkit (GATK)	Broad Institute	https://gatk.broadinstitute.org/hc/en-us
ANNOVAR	Wang et al.^29^	https://annovar.openbioinformatics.org/en/latest/
BEAST	Bouckaert et al.^30^	https://beast.community/
FigTree	University of Edinburgh	https://tree.bio.ed.ac.uk/software/figtree/
R statistical environment	R Foundation	https://www.r-project.org/
fgsea package	Korotkevich et al.^31^	https://bioconductor.org/packages/release/bioc/html/fgsea.html
MsigDB Hallmark and KEGG gene sets	Liberzon et al.^32^	https://www.gsea-msigdb.org/gsea/msigdb
SigProfilerExtractor	Islam et al.^5^	https://github.com/AlexandrovLab/SigProfilerExtractor
ClinVar database	Landrum et al.^33^	https://www.ncbi.nlm.nih.gov/clinvar/
MetaSVM algorithm	Liu et al.^34^	https://data.igvf.org/software/metasvm/
COSMIC mutational signature database	Sondka et al.^35^	https://www.sanger.ac.uk/tool/cosmic/

**Other**		

Custom code	This paper	https://doi.org/10.5281/zenodo.17675928

### Experimental model and study participant details

#### Patient population

Patients followed at the Yale Cancer Prevention Clinic (YCPC) were selected for this study if they had breast cancer at or before age 50, had a high-risk family history followed by the National Comprehensive Cancer Network (NCCN) guidelines,^36^ and were negative for high-penetrance germline mutations in 23 known breast cancer-predisposing genes assessed by the Ambry Genetics BRCANext-Expanded assay. All participants signed informed consent for germline DNA analysis and this study was approved by the Yale Institutional Review Board.

Ninety-four women met eligibility criteria; germline DNA from peripheral white blood cells was available for all patients, and 36 patients also had primary breast cancer tissue available from surgical resections for genomic analysis. All tissue samples were reviewed by a board-certified pathologist, who identified and marked the areas of histologically normal breast epithelium located at the greatest possible distance from the tumor mass within the available specimen. Twenty-four patients had all three sample types available (white blood cells for germline DNA, breast cancer, and cancer-adjacent normal breast tissues). The blood samples were stored at −80°C until DNA extraction and all breast tissue samples were formalin-fixed and paraffin-embedded (FFPE). All but two cancers were estrogen hormone receptor-positive (HR+), one was Human Epidermal Growth Factor Receptor-2 positive (HER2+)/HR-, and one was triple-negative (TNBC).

We also studied a separate young breast cancer patient cohort from The Cancer Genome Atlas (TCGA), dbGaP: phs000178.v11.p8. Seventeen patients (11 ER+/HER2-, 3 HER2+/ER-, 3 TNBC) were selected based on the following criteria: the patient had WES data from matching blood, normal, and tumor tissue samples and was diagnosed with breast cancer at an age younger than 50. Sequencing data from TCGA were downloaded in.bam file format.

Gender distribution was limited to female participants, reflecting the disease’s prevalence. Socioeconomic and ancestry data were not available.

### Method details

#### Whole-exome sequencing and mutation calling

DNA was extracted using the Qiagen All Prep Universal kit. DNA sequencing was performed at the Yale Center for Genome Analysis. The quality and quantity of DNA were tested on the Agilent 2100 Bioanalyzer system. FFPE DNA samples mixed with Random Primer, incubated for 5 min at 37°C followed by the addition of NEBNext FFPE DNA Repair Mix (New England BioLab, cat# M6630L) and incubation for 15 min at 20°C. DNA was sheared to a mean fragment length of 220 bp using the Covaris E210 instrument, purified by Magnetic AMPure XP Beads (Beckman Coulter), and labeled with a 6-bp barcode during PCR amplification. Exomes were captured using the IDT xGen Exome Research Panel v1.0 and libraries were sequenced on Illumina HS4000 instrument using 100-base pair paired-end reads by multiplexing four tumor samples per lane. The coverage for blood and normal adjacent was ∼100×, while for the tumor, it was ∼200×. The same DNA extraction and sequencing methods were used for blood, tumor, and normal adjacent breast tissue samples.

The TCGA samples had a similar number of reads, mean coverage, and PCR duplicates across the tissue types. In the Yale dataset, cancers had twice the number of reads and mean coverage as blood and cancer-adjacent normal breast tissues (Figure S1A). The age distributions (Figure S1B) were similar in both cohorts (23–49 years, median 43).

Variant calling was performed in the same way for both the TCGA and Yale samples. Sequences were mapped to the human reference genome vGRCh38 using Burrows-Wheeler Aligner (v0.7.15), PCR duplicates were marked with Picard (v2.17.11), and the IndelRealigner and RealignerTargetCreator from GATK (v3.4) were used to adjust the alignment of indel regions. GATK was also used for base quality score recalibration. Germline single nucleotide mutations and indels for each sample were called using Haplotype Caller. GVCF files were consolidated, and the GenotypeGVCFs tool was used for the joint-calling of variants. The resulting variants were filtered based on quality and applied to hard-filtering using specified thresholds for single nucleotide mutations: DP < 4, QD < 2, FS > 60, MQ < 35, MQRankSum < −12.5, ReadPosRankSum < −8, and for indels: DP < 4, QD < 2, FS > 200, MQ < 35, ReadPosRankSum < −20. The remaining variants were annotated with Annovar.^29^ The final VCF file was filtered (GQ < 20, removal of intron variants) to contain only mutations in coding regions.

Not all patients had complete sets of three sequencing data due to a lack of adequate tissue or file corruption (Table S1). In the Yale cohort, blood samples were available from all patients; eight patients had blood and tumor samples, and four patients had blood and cancer-adjacent normal samples. In the TCGA cohort, one patient had only blood and tumor data. In the final analysis, we only included cases that had a full set of matching blood, cancer-adjacent normal, and cancer data (Yale cohort *n* = 24, TCGA cohort *n* = 17).

Somatic mutations were defined as variants detected in cancer or cancer-adjacent normal tissues, but not in matching blood. Venn diagrams at the gene level were generated along with the Jaccard index to compare variants between blood, cancer-adjacent normal, and tumor tissue.

#### Phylogenetics analysis

For each Yale case with matched blood, cancer-adjacent normal, and tumor WES, we built a per-patient somatic variant matrix. To reduce false-negative calls from uneven depth, we re-evaluated read counts at candidate loci with a coverage-aware multinomial variant-calling procedure as described by Zhao et al.^37^ The validated, jointly covered loci were concatenated into an alignment and used to infer per-patient tumor phylogenies. We estimated phylogenies in BEAST v2.7.7^30^ under a GTR substitution model with a relaxed molecular clock. After burn-in, three MCMC chains were merged with LogCombiner and summarized to maximum-clade-credibility trees in TreeAnnotator. Trees, posterior node supports and branch lengths were visualized in FigTree. Tumor expansion was inferred from the structure and branch length in trees, enabling assessment of clonal evolution and evaluation of claims of field cancerization.

### Quantification and statistical analysis

To obtain high functional impact variants, raw variants from each sample were filtered using the R programming language (version 4.2.2.). Only mutations predicted as deleterious/pathogenic variants by the MetaSVM^34^ ensemble algorithm or by ClinVar^33^ were included in the analysis and are referred to as high functional impact. Fisher’s exact test was used to determine statistical significance of differentially mutated genes between pairs of tissues, with Benjamini & Hochberg (BH) adjustment for multiple testing and Cramer’s V effect size.

To address the question of whether the number of somatic variants increases with age, we calculated Pearson’s correlation coefficient between age and Mutation Burden (MB) - which was defined as the total number of variants in a gene divided by the total length of the gene in megabases.

Mutational differences at the biological pathway level between tissues were assessed using the fgsea package^31^ in R for Gene Set Enrichment Analysis (GSEA) using the MsigDB Cancer Hallmarks^38^ and KEGG C2 Cancer Pathway^32^ gene sets. The log odds ratio was calculated over reference tissue (blood vs. normal, blood vs. tumor, normal vs. tumor) to rank the genes for GSEA, and both with the Normalized Enrichment Scores (NES) were provided as standardized effect sizes. Statistical significance shown in the figures (asterisks) corresponds to GSEA-derived enrichment significance based on permutation testing (FDR q-values). For a manually curated list of 1,558 cancer hallmark genes that were assigned to 21 pathways,^5^ the average percent of pathway member genes affected by high functional impact germline or somatic variants was also calculated to show pathway-level disturbance from somatic and germline origin.

The SigProfilerExtractor^39^ was employed for mutational signature analysis in both cohorts and each tissue type. The tool enabled the precise identification and extraction of mutational patterns. The signatures were decomposed into the known COSMIC single base substitution (SBS) signatures^35^ for a better understanding of mutational processes active in breast cancer. The Wilcoxon signed-rank test was performed to investigate the differences between cohorts in the resulting mutation type probabilities.

## Supplemental information

Document S1. Figures S1–S7

Table S1. Details of Yale and TCGA cohorts: Patients and samples used in the study, related to Table 1

Table S2. Genes mutated only in tissues of Yale cohort patients: 0 = no mutation, 1 = mutation, related to Figure 2 The table includes results from differential expression analysis in TCGA between tumor and normal tissues for verification of the variants’ expression: log2 fold change and adjusted *p* values.

Table S3. Genes mutated only in tumor tissue of TCGA cohort patients, related to Figure 2 0 = no mutation, 1 = mutation.

Table S4. Jaccard index based on Venn diagrams at a gene level (Figure S2) between blood & normal samples and normal & tumor samples for Yale and TCGA cohorts, related to Figure 2

Table S5. Details of shared somatic mutations between tumor and normal adjacent tissues in Yale cohort, related to Figure 5

Table S6. Fisher’s exact test results for differentially mutated genes in the Yale cohort, related to Figure 3

Table S7. Fisher’s exact test results for differentially mutated genes in the TCGA cohort, related to Figure 3

Document S2. Article plus supplemental information
