# Supplementary material for: Mutational landscape of normal breast tissues adjacent to invasive breast cancer
Source: Cell Rep Med. 2026 Jan 8;7(1):102543. doi: 10.1016/j.xcrm.2025.102543 (PMC12866091; doi:10.1016/j.xcrm.2025.102543)
Supplement: Document S1. Figures S1–S7 [file mmc1.pdf]

Cell Reports Medicine, Volume *7*

Supplemental information

Mutational landscape of normal breast tissues adjacent to invasive breast cancer

Aleksandra Suwalska, Mariya Rozenblit, Malini Harigopal, Jiawei Dai, Meng Liu, Jeffrey P. Townsend, Michal Marczyk, and Lajos Pusztai

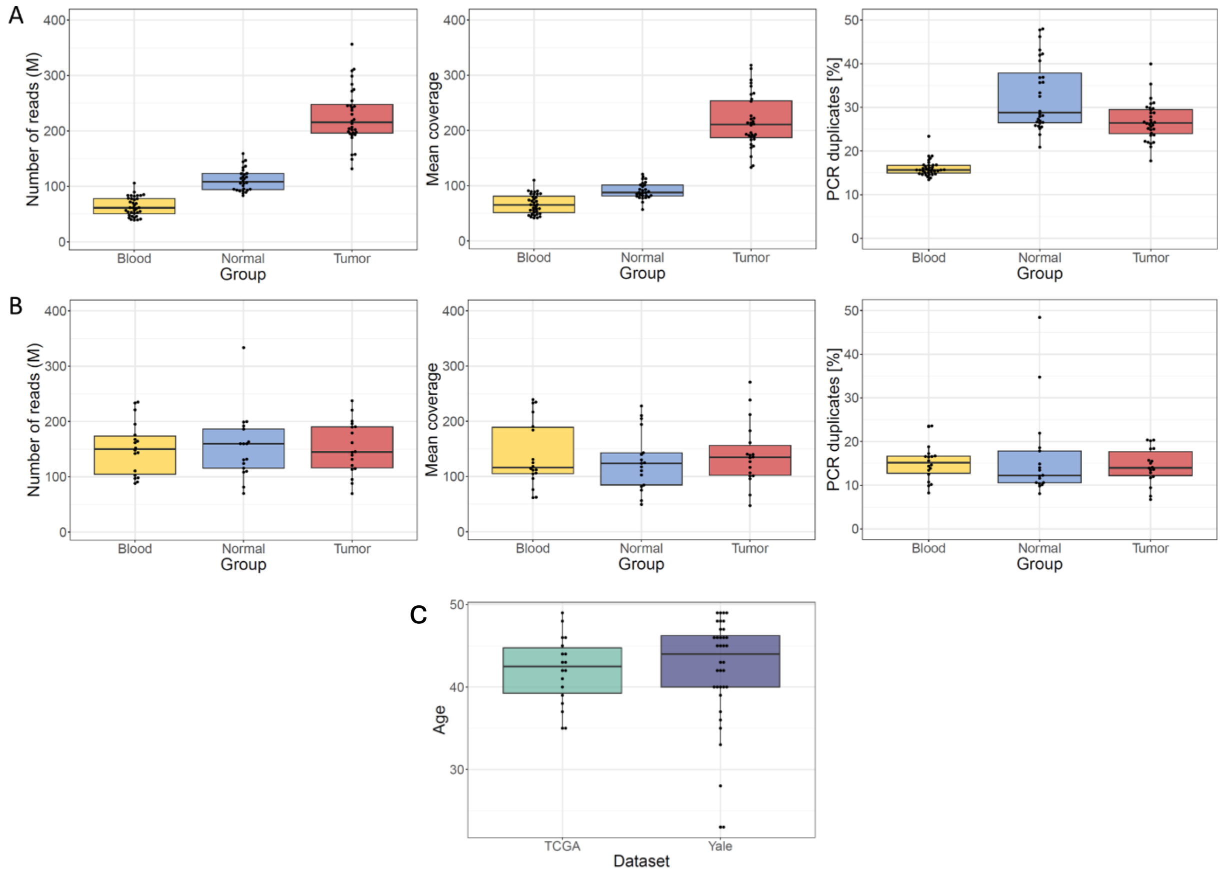

Figure S1. Quality metrics and demographic characteristics across Yale and TCGA cohorts. A.) Comparison of the number of reads (M), mean coverage, and PCR duplicates between tissues within Yale cohort. B.) Comparison of the number of reads (M), mean coverage, and PCR duplicates between tissues within TCGA cohort. C.) Age distribution in TCGA and Yale cohorts. Related to Table 1.

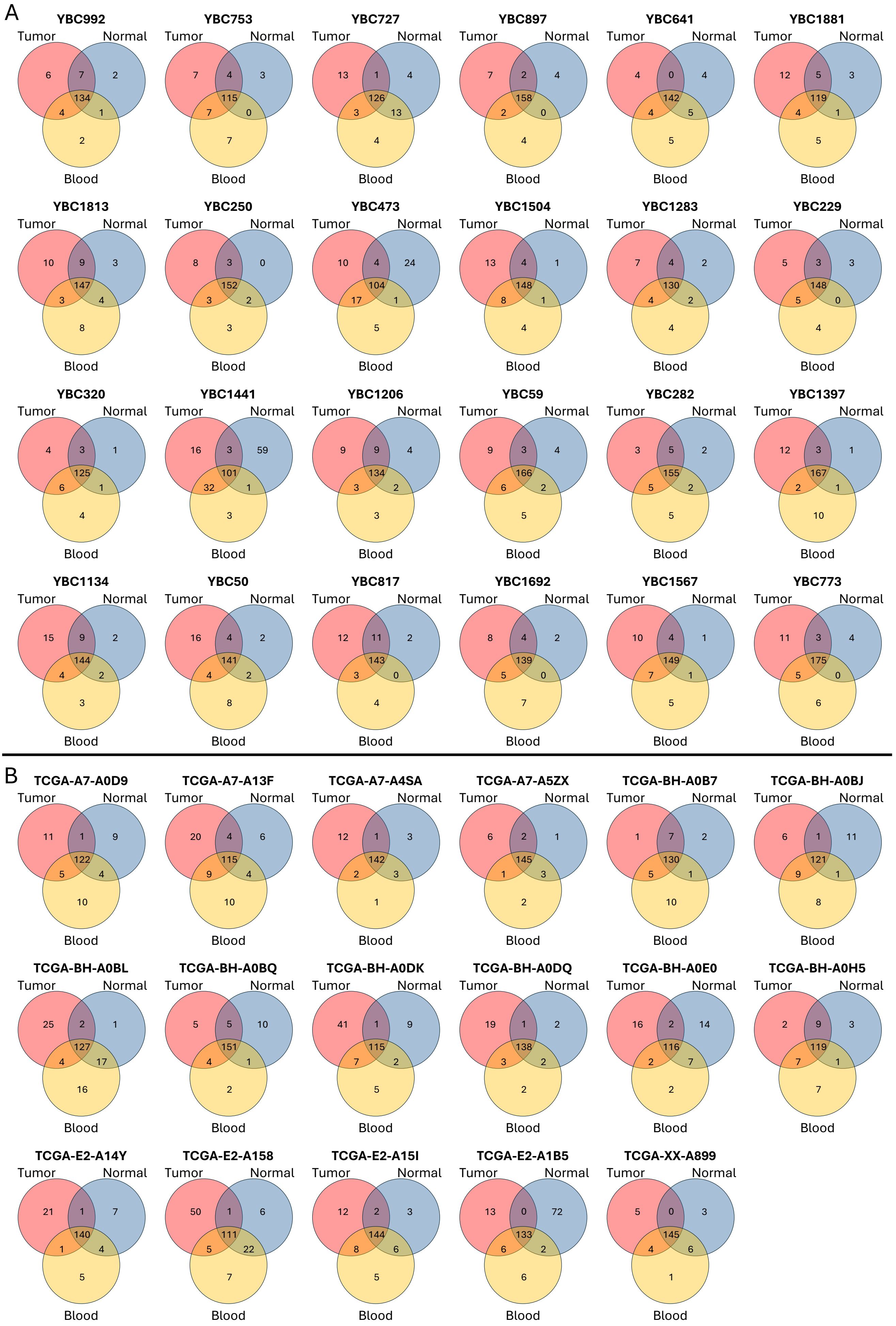

Figure S2. Venn diagrams at a gene level for patients with three matching tissue samples. A.) Yale cohort. B.) TCGA cohort. Related to Figure 2.

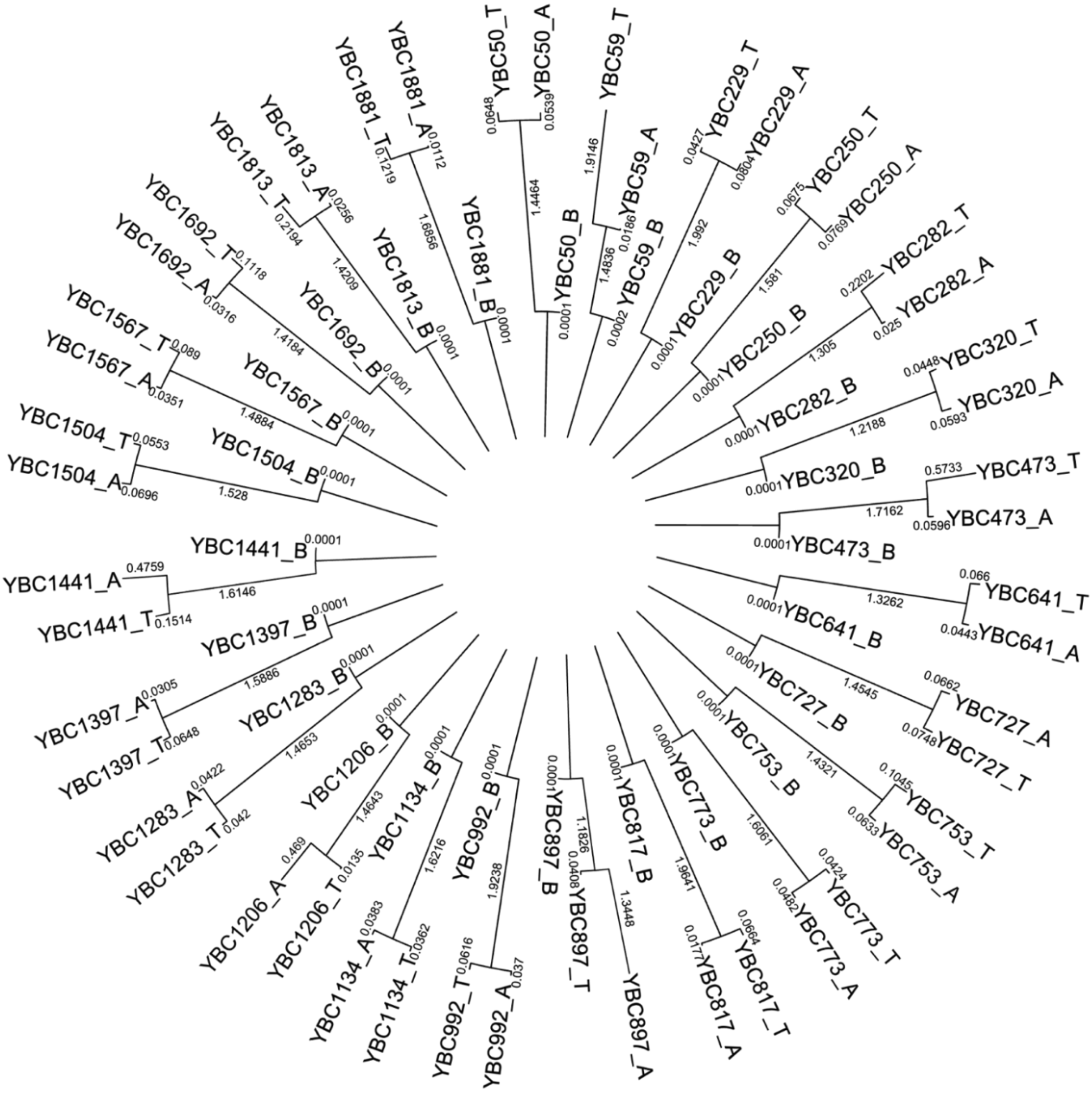

Figure S3. Phylograms of 24 lung cancer patients inferred from BEAST2 MCC trees. Each phylogram represents the mutation-scaled evolutionary relationship among tumor (T), adjacent normal (A), and blood (B) samples from an individual patient. Branch lengths, shown on the branches, correspond to the expected number of substitutions per site, calculated as the product of the evolutionary rate and time inferred from BEAST2. Trees are displayed radially around a circular layout for visualization, with the blood (B) sample positioned toward the inner circle to represent the germline root, and tumor (T) and adjacent normal (A) samples extending outward according to their evolutionary divergence. Related to Figure 1.

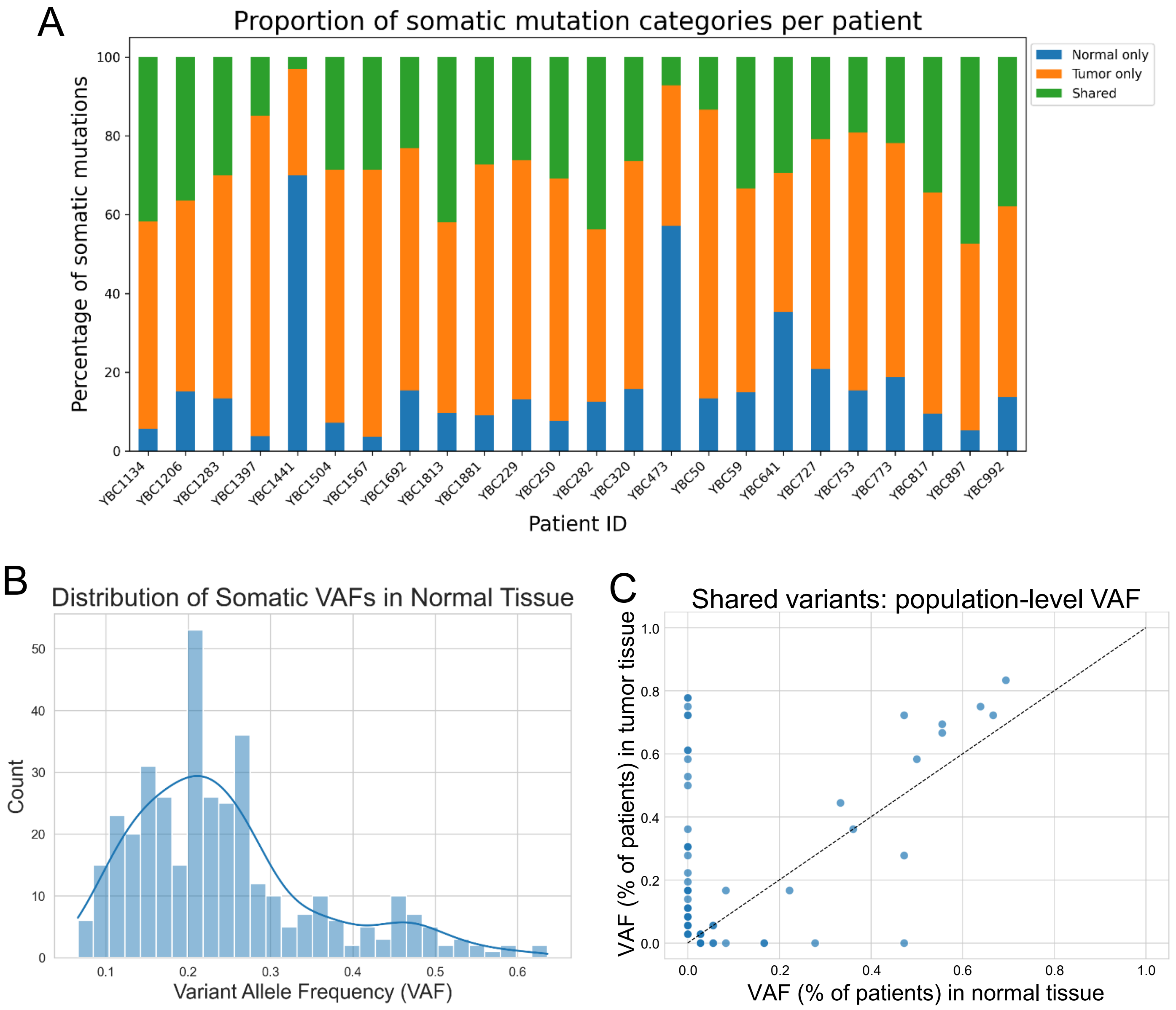

Figure S4. Distribution and characteristics of somatic mutations in tumor and normal tissue adjacent to breast tumor. A.) The proportion of somatic mutations that are private to the tumor (orange), private to adjacent normal tissue (blue), or shared between both tissues (green) for each patient in the Yale cohort. The analysis was restricted to somatic mutations not found in matched blood samples. B.) Variant Allele Frequency distribution of somatic variants for normal tissue adjacent to the tumor. C.) Comparison of variant allele frequencies (as the fraction of patients) of shared somatic mutations in adjacent normal (x-axis) and tumor (y-axis) tissues in the Yale cohort. Related to Figure 5.

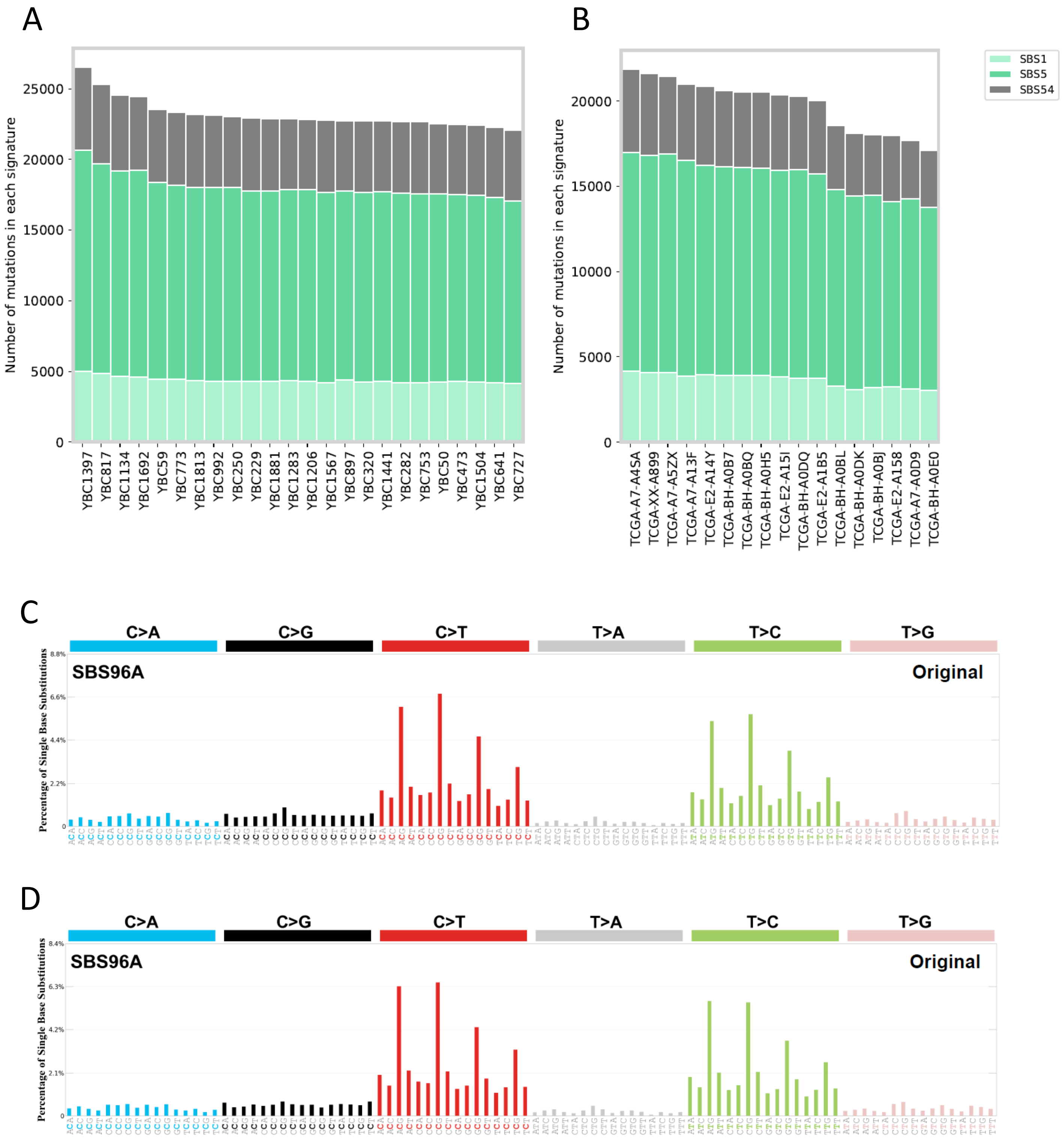

Figure S5. Mutational signature analysis results. Activity plots – contribution of each identified mutational signature within each sample analyzed. A.) Yale tumor samples. B.) TCGA tumor samples. C.) De novo mutational signature for Yale tumor samples. D.) de novo mutational signature for TCGA tumor samples. Related to Table 1.

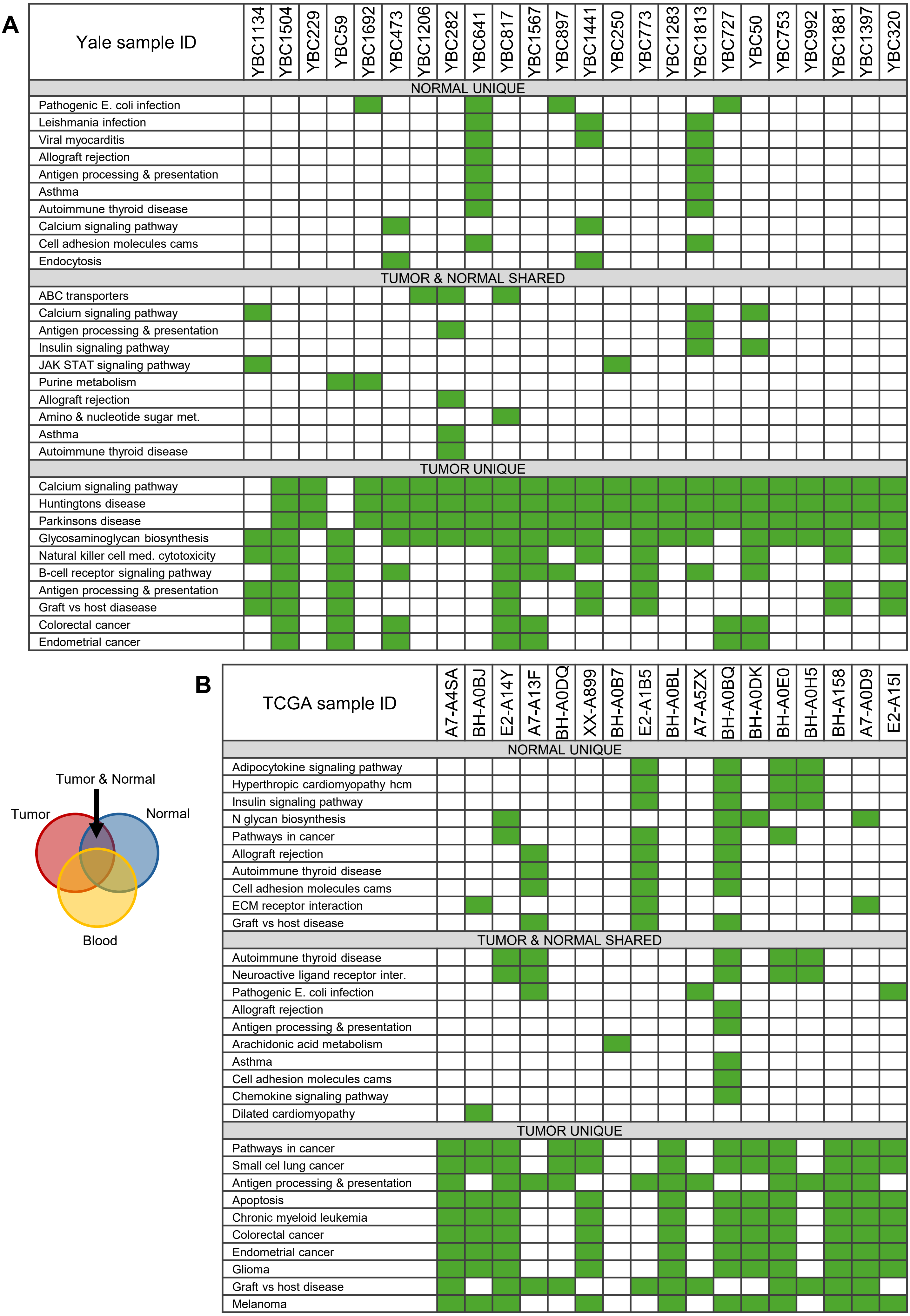

Figure S6. Affected KEGG pathways by the mutations in normal and tumor tissues. Each column is a different patient indicated with a unique ID, and each row is a pathway affected by genes unique to the normal or tumor tissues or shared between them. The green color indicates the affected KEGG pathway. A.) Yale cohort. B.) TCGA cohort. Related to Figure 4.

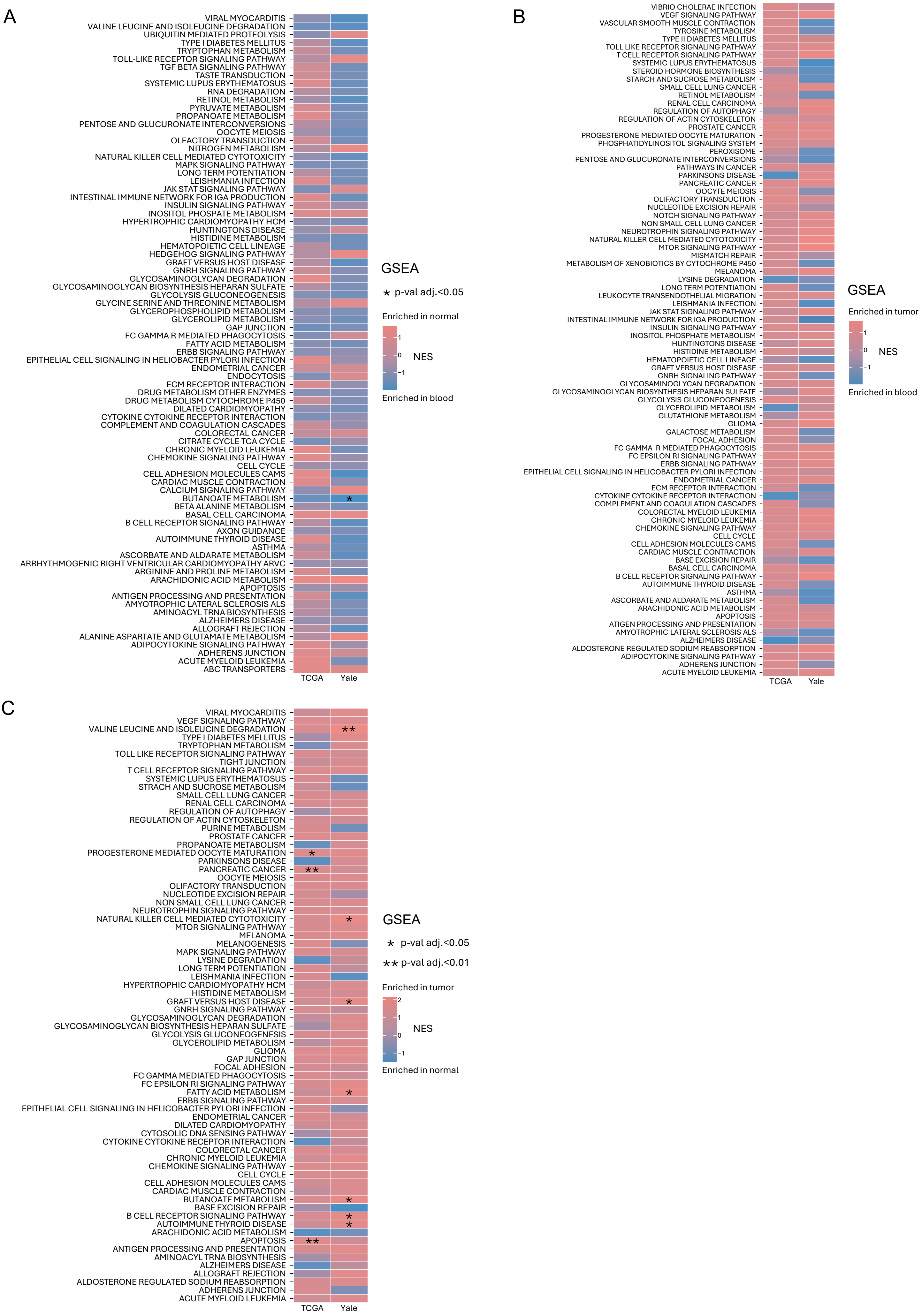

Figure S7. Gene Set Enrichment Analysis results for the KEGG pathways subset. The Normalized Enrichment Scores (NES) were provided as standardized effect sizes. Statistical significance shown in the figures (asterisks) corresponds to GSEA-derived enrichment significance based on permutation testing (FDR q-values). A.) Blood vs normal tissue. B.) Blood vs tumor tissue. C.) Normal vs tumor tissue. Related to Figure 4.
